# Seroprevalence and Determinants of Hepatitis B Virus and Hepatitis C Virus Infection Among Pregnant Women in North India: A Cross-Sectional Study

**DOI:** 10.7759/cureus.101805

**Published:** 2026-01-18

**Authors:** Abhishek Yadav, Monika Agarwal, Vikasendu Agarwal, Amita Jain, Sumit Rungta, Prabhaker Mishra, Amit Goel, Anjoo Agarwal, Himanshu Reddy, Priyanka Yadav, Milind Wardhan, Neeraj K Gupta, Manisha Pathak

**Affiliations:** 1 Community Medicine and Public Health, King George's Medical University, Lucknow, IND; 2 Medical and Health Services, State Viral Hepatitis Programme Management Unit, National Viral Hepatitis Control Programme (NVHCP), Lucknow, IND; 3 Microbiology, All India Institute of Medical Sciences, Raebareli, IND; 4 Gastroenterology, King George's Medical University, Lucknow, IND; 5 Biostatistics and Health Informatics, Sanjay Gandhi Postgraduate Institute of Medical Sciences, Lucknow, IND; 6 Gastroenterology, Sanjay Gandhi Postgraduate Institute of Medical Sciences, Lucknow, IND; 7 Obstetrics and Gynaecology, King George's Medical University, Lucknow, IND; 8 Internal Medicine, King George's Medical University, Lucknow, IND; 9 Obstetrics and Gynaecology, Silver Jubilee Community Health Centre (CHC), Lucknow, IND; 10 Child Health Division, National Health Mission, Lucknow, IND; 11 Family Medicine, King George's Medical University, Lucknow, IND; 12 Public Health, Sarsawati Medical College, Lucknow, IND

**Keywords:** hepatitis c viral infection, india, maternal screening, model treatment centre, neonatal immunoprophylaxis, pregnancy, vertical transmission, viral hepatitis b

## Abstract

Background

Maternal hepatitis B virus (HBV) and hepatitis C virus (HCV) infections remain major contributors to perinatal and early childhood hepatitis transmission in India. Despite national guidelines recommending universal antenatal screening, coverage gaps persist. This study aimed to determine the prevalence, associated risk factors, and preventive outcomes of HBV and HCV among pregnant women in Lucknow District, North India.

Objectives

The primary objectives of this cross-sectional study were to determine the prevalence of HBV and HCV among pregnant women attending antenatal clinics (ANCs) at primary and secondary health facilities in Lucknow; to assess the risk factors associated with HBV and HCV infection among pregnant women; and to study pregnancy outcomes among HBV/HCV-positive pregnant women. Additionally, we documented whether neonates born to HBV-positive mothers received standard immunoprophylaxis, including hepatitis B immunoglobulin (HBIG) and the hepatitis B birth-dose vaccine.

Methods

A hospital-based cross-sectional study was conducted among 2005 pregnant women attending ANCs. Serum samples were tested for hepatitis B surface antigen (HBsAg) and anti-HCV antibodies using rapid diagnostic kits at the health facilities, followed by viral load assessment at the Model Treatment Centre (MTC). Sociodemographic, obstetric, and exposure-related data were analyzed using univariate and multivariate logistic regression to identify independent predictors. The timely administration of the hepatitis B birth-dose vaccine and HBIG to neonates born to hepatitis-positive mothers, in accordance with established national protocols, was also documented.

Results

The overall prevalence of viral hepatitis among pregnant women was 2.8%, including 2.5% HBsAg positivity and 0.3% anti-HCV positivity, with no observed co-infection. Prevalence was significantly higher among women aged ≥25 years. Independent predictors of infection included a sibling history of hepatitis (adjusted odds ratio (AOR) 11.68, p=0.001), unsafe injections administered by unqualified practitioners (AOR 5.07, p=0.024), sharing of sharp instruments such as blades or razors (AOR 5.58, p=0.018), a history of jaundice or prior HBsAg positivity (AOR 5.93, p=0.021), and a family member receiving treatment for HBV or HCV (AOR 15.36, p=0.003). Maternal clinical parameters were reviewed to facilitate appropriate referral and follow-up care in accordance with ethical guidelines.

Conclusion

This study underscores the importance of universal antenatal screening and regulation of unsafe injection practices. The implementation of comprehensive maternal screening in conjunction with timely neonatal immunoprophylaxis may contribute to reducing vertical transmission and aligns with India’s commitment to the World Health Organization’s 2030 hepatitis elimination agenda.

## Introduction

Viral hepatitis caused by the hepatitis B virus (HBV) and the hepatitis C virus (HCV) continues to impose a substantial burden on global health systems, with an estimated 354 million people living with chronic infection worldwide [1]. HBV remains endemic in many parts of Asia and Africa, where transmission occurs through both perinatal and non-perinatal routes, sustaining long-term community spread [2]. Within India, the prevalence of HBV infection differs markedly across regions and populations, with reported rates ranging between 2% and 7% [3]. Persistent HBV infection is a major contributor to advanced liver disease, including cirrhosis and hepatocellular carcinoma, and accounts for approximately 820,000 deaths annually on a global scale [1]. Although HCV infection is comparatively less common, its clinical importance is underscored by the high likelihood of chronicity, which develops in more than half of infected individuals and may lead to progressive liver damage [4].

In the context of pregnancy, HBV and HCV infections raise additional concerns due to the risk of vertical transmission, unrecognized chronic disease in mothers, and potential long-term health consequences for exposed infants. In India, national policies advocate routine antenatal screening for HBV and selective screening for HCV; however, the availability of updated and district-level epidemiological data remains inconsistent. As a result, the true burden of maternal viral hepatitis and the effectiveness of preventive measures are not uniformly well characterized.

Assessing local prevalence in high-volume obstetric care settings is essential for informed clinical decision-making and public health planning. Previous studies from Uttar Pradesh have reported variable rates of HBV and HCV seropositivity among pregnant women, but information regarding associated risk factors and adherence to recommended preventive interventions remains limited.

The present study was designed to provide updated, hospital-based estimates of HBV and HCV prevalence among pregnant women attending antenatal services in Lucknow, Uttar Pradesh. In addition, maternal characteristics potentially linked to blood-borne viral exposure-such as family history of hepatitis, prior medical or dental procedures, and exposure to unsafe injections-were evaluated. The administration of neonatal immunoprophylaxis following identification of maternal HBV infection was recorded descriptively to reflect prevailing clinical practices.

Evidence from India and other low- and middle-income countries indicates that suboptimal vaccination coverage, limited awareness, and unsafe healthcare practices continue to drive hepatitis transmission [5]. During pregnancy, viral hepatitis infections may increase the risk of maternal complications and facilitate perinatal transmission, with lasting implications for infant health [6]. Despite the availability of effective vaccines for HBV and highly efficacious antiviral therapies for HCV, gaps persist in screening uptake, timely immunoprophylaxis, and linkage to care among pregnant women in India [6,7].

The World Health Organization has identified viral hepatitis elimination as a priority public health goal, targeting a substantial reduction in new infections and hepatitis-related mortality by 2030 [1,8]. Antenatal screening and prompt neonatal vaccination play a central role in achieving this objective. Although pregnancy does not typically worsen liver disease activity, careful monitoring is required to mitigate the risk of mother-to-child transmission [9]. Current recommendations advise that infants born to HBV-positive mothers receive hepatitis B immunoglobulin (HBIG) and a birth-dose hepatitis B vaccine within the first 24 hours of life, in accordance with World Health Organization and National Viral Hepatitis Control Programme guidelines [10,11].

Against this background, the present study aims to estimate the prevalence and determinants of HBV and HCV among pregnant women in Lucknow, Uttar Pradesh. The findings may help inform local public health interventions and support national and global hepatitis elimination efforts.

## Materials and methods

Study design and setting

A hospital-based cross-sectional study was conducted from January 2023 to December 2024 (a period of 2 years) across selected primary and secondary health care facilities in Lucknow district, Uttar Pradesh, India. To ensure adequate representation of both urban and rural populations, health care facilities were selected using a systematic random sampling approach, which included two urban primary health care facilities out of nine urban health facilities and two rural primary health care facilities out of nine rural health care facilities.

Sampling technique and sample size

Systematic random sampling was employed to recruit pregnant women attending antenatal clinics (ANCs) at the selected health care facilities after rapid diagnostic screening using J. Mitra rapid diagnostic kits for HBsAg and HCV. Based on the estimated antenatal attendance during the study period, the sampling interval (k) was calculated as 3. After selecting a random starting point using the lottery method, every third eligible pregnant woman was enrolled in the study. This sampling process was implemented uniformly across all selected urban and rural health care facilities until the predetermined sample size of 2005 pregnant women was achieved, ensuring representative coverage and minimizing selection bias.

Participant enrollment and laboratory procedures

A total of 2005 pregnant women attending ANCs were enrolled using systematic random sampling, wherein every third eligible woman presenting for antenatal care was invited to participate after obtaining written informed consent. Pregnant women with a known history of chronic liver disease and those who declined participation were excluded from the study. Women who declined blood testing or did not provide consent were also excluded.

All enrolled participants were initially screened for hepatitis B surface antigen (HBsAg) and anti-hepatitis C virus (anti-HCV) antibodies using J. Mitra rapid diagnostic test kits. For women who tested positive, 5 mL of venous blood was collected and transported to the Department of Microbiology, King George’s Medical University (KGMU), Lucknow, a designated reference laboratory under the NVHCP, for confirmatory testing and viral load estimation.

Serological testing was performed using enzyme-linked immunosorbent assay (ELISA) kits manufactured by J. Mitra & Co. Pvt. Ltd., New Delhi, India. Detection of HBsAg was carried out using the Hepalisa Ultra (4th generation) ELISA, based on the direct sandwich ELISA principle and employing monoclonal antibodies for antigen capture and detection. The assay has a reported analytical sensitivity of approximately 0.05 ng/mL, with high diagnostic sensitivity and specificity as per manufacturer data. Detection of anti-HCV antibodies was performed using the HCV Microlisa (3rd generation) ELISA, which follows an indirect ELISA principle utilizing multiple recombinant HCV antigens (Core, E1, E2, NS3, NS4, and NS5). All assays were performed according to the manufacturer's instructions with appropriate internal quality controls.

Clinical referral and follow-up

Pregnant women with confirmed HBV or HCV infection were classified as high-risk pregnancies and referred to tertiary care centers for appropriate antenatal management and delivery planning. Infants born to hepatitis B-positive mothers received HBIG and the birth-dose hepatitis B vaccine within 24 hours of birth, in accordance with WHO and NVHCP guidelines, which was documented. Spouses of infected women were counseled and offered screening; those found positive were referred to the Model Treatment Centre (MTC) at King George’s Medical University (KGMU), Lucknow, for further evaluation and management. Additionally, HBV- and HCV-positive pregnant women were linked to the Department of Gastroenterology, KGMU, Lucknow, for specialized care and follow-up.

Data collection and definitions

Data collection and definitions: Data were collected using a structured questionnaire. Operational definitions were applied for key exposures, including unsafe injections (receipt of injections from informal or unqualified practitioners, as self-reported), invasive procedures (history of surgeries, dental work, or blood transfusions), and family history of hepatitis (known history of HBV or HCV in first-degree relatives). Self-reported exposures are acknowledged as being subject to recall bias and misclassification.

Statistical analysis

Descriptive statistics were used to summarize the sociodemographic, clinical, and obstetric characteristics of the participants. Categorical variables are presented as numbers (%) and quantitative variables as mean ± standard deviation. Associations between independent variables and HBV positivity were initially examined using the chi-square test (univariate analysis), and variables with a p-value <0.05 were subsequently included in a multivariable binary logistic regression model to adjust for potential confounding factors. A penalized logistic regression approach was used due to the inclusion of multiple variables, leading to improved predictive performance and feature selection.

Before multivariable analysis, multicollinearity was assessed using the variance inflation factor (VIF), with values <5 considered acceptable. Model adequacy was evaluated using appropriate goodness-of-fit measures. Missing data were assessed and handled using complete-case analysis. Results are presented as adjusted odds ratios (AORs) with 95% confidence intervals (CIs), with cautious interpretation due to potential model instability.

Data were entered and analyzed using the Statistical Package for the Social Sciences (SPSS) software, version 27 (IBM, Chicago, IL, USA). A p-value <0.05 was considered statistically significant.

Ethical approval

The study received approval from the Institutional Ethics Committee of King George’s Medical University, Lucknow (Approval No. 121st ECMII B-Ph.D/PI; dated August 16, 2023). A statement confirming that written informed consent was obtained from all participants has also been included.

## Results

Table 1 presents the association between sociodemographic and obstetric factors with HBV and HCV infection. The prevalence of infection was higher among women aged ≥25 years (3.56%) compared to those aged <25 years (1.76%), and this difference was statistically significant (p=0.0159). Similarly, urban women showed a slightly higher infection rate (3.02%) than rural women (2.28%; p=0.35). Literate women were associated with a marginally higher prevalence (2.91%) compared to Women with no formal education (2.11%; p=0.44). Women from low-income households (<₹20000/month) showed an infection prevalence of 0.95%, while those earning ≥₹20000/month had a prevalence of 9.8% (p<0.001), suggesting socioeconomic disparity. Prevalence estimates are presented descriptively. Income-related associations should be interpreted with caution due to potential confounding and selection effects. By obstetric profile, infection was highest in the first trimester (3.95%), followed by the Second (3.58%) and third trimester (2.03%), however, this difference was not statistically significant (p=0.078). Primigravida women had slightly higher infection rates (3.12%) than multigravida (2.57%; p=0.46).

**Table 1:** Relationship between sociodemographic and obstetric characteristics and hepatitis infection among the study participants HCV: hepatitis C virus; HBV: hepatitis B virus; CI: confidence interval

Characteristics	Total (n)	Total (%)	With HBV/HCV (n)	With HBV/HCV (%)	Without HBV/HCV (n)	Without HBV/HCV (%)	Prevalence % (95% CI)	P-value
Age group (years)								
<25 years	852	42.5	15	1.76	837	98.24	1.76 (0.96-2.89)	0.0159
≥25 years	1153	57.5	41	3.56	1112	96.44	3.56 (2.59-4.80)	
Residence								
Rural	615	30.7	14	2.28	601	97.72	2.28 (1.26-3.80)	0.35
Urban (+slum)	1390	69.3	42	3.02	1348	96.98	3.02 (2.18-4.05)	
Religion								
Hindu	1042	52.0	40	3.84	1002	96.16	3.84 (2.76-5.21)	0.0031
Muslim+others	963	48.0	16	1.66	947	98.34	1.66 (0.95-2.68)	
Educational status								
No formal education	285	14.2	6	2.11	279	97.89	2.11 (0.77-4.54)	0.44
Literate	1720	85.8	50	2.91	1670	97.09	2.91 (2.16-3.82)	
Occupation								
Housewife	1951	97.3	52	2.67	1899	97.33	2.67 (2.00-3.48)	0.037
Employed	54	2.7	4	7.41	50	92.59	7.41 (2.05-17.99)	
Monthly family income (₹)								
<20000	1587	79.1	15	0.95	1572	99.05	0.95 (0.53-1.57)	<0.001
≥20000	418	20.9	41	9.81	377	90.19	9.81 (7.09-13.03)	
Gestational age (weeks)								
1st trimester	253	12.6	10	3.95	243	96.05	3.95 (1.91-7.14)	0.078
2nd trimester	670	33.4	24	3.58	646	96.42	3.58 (2.30-5.28)	
3rd trimester	1082	54.0	22	2.03	1060	97.97	2.03 (1.28-3.04)	
Gravida								
Primigravida	801	39.9	25	3.12	776	96.88	3.12 (2.02-4.58)	0.46
Multigravida (≥2)	1204	60.1	31	2.57	1173	97.43	2.57 (1.75-3.62)	
Total	2005	100	56	2.8	1949	97.2	-	-

Tables 2, 3, 4 present the prevalence and regression analyses among a total of 2005 pregnant women. The overall prevalence of viral hepatitis was 2.8%, with 2.5% (n=50) testing positive for HBsAg and 0.3% (n=6) testing positive for anti-HCV. No cases of HBV-HCV coinfection were observed. The majority of women were aged 25-30 years (57.5%) and resided in urban areas (69.3%). Most participants were literate (85.8%) and homemakers (97.3%), with nearly three-fourths reporting a monthly family income below ₹20000. Obstetric profiling revealed that 54% were in the third trimester and 60% were multigravida. Univariate (unadjusted) logistic regression analysis showed that several behavioral and clinical factors were not significantly associated with HBV or HCV infection. For example, multiple ear or nose piercings (odds ratio (OR): 0.55, 95% CI: 0.20-1.54, p=0.254), permanent tattooing (OR: 0.83, 95% CI: 0.26-2.70, p=0.759), and injectable needle injuries (OR: 3.85, 95% CI: 0.48-30.89, p=0.205) showed no significant association. Higher unadjusted odds were observed for a history of blood transfusion, dialysis, and minor or major surgery, although many of these associations did not reach statistical significance.

**Table 2:** Prevalence of hepatitis (HBV and HCV) among pregnant women HCV: hepatitis C virus; HBV: hepatitis B virus; HBsAg: hepatitis B surface antigen

Infection	Prevalence (%)	Number
HBV (HBsAg+)	2.50%	50
HCV (anti-HCV+)	0.30%	6
Overall positive	2.80%	56

**Table 3:** Prevalence of hepatitis (HBV and HCV) by trimester among pregnant women HCV: hepatitis C virus; HBV: hepatitis B virus; CI: confidence interval

Trimester	HBV (n)	(%)	HCV (n)	(%)	Total positive (n)	(%)	95% CI (lower-upper)
1^st ^(n=253)	10	-0.60%	0	0	10	-0.60%	2.16-7.12
2^nd^ (n=670)	21	-1.00%	3	-0.15%	24	-1.15%	2.42-5.27
3^rd^ (n=1082)	19	-0.90%	3	-0.15%	22	-1.05%	1.29-2.94

**Table 4:** Multivariable logistic regression of risk factors for HBV/HCV infection among pregnant women (total n=2005; hepatitis-positive women n=56) HCV: hepatitis C virus; HBV: hepatitis B virus; HBsAg: hepatitis B surface antigen; OR: odds ratio

Variable	Unadjusted OR (95% CI)	Adjusted OR (95% CI)	P-value (adjusted)
Sharing sharp materials (blades/razors) for the removal of body hairs	5.22 (1.34-14.98)	5.58 (1.29-15.10)	0.018
History of jaundice/prior HBsAg positivity	6.22 (1.35-28.55)	5.93 (1.17-30.08)	0.021
Any sibling diagnosed with HBV/HCV	10.24 (2.55-39.11)	11.68 (2.77-42.97)	0.001
Unsafe injections from informal healthcare providers	4.85 (1.10-6.25)	5.07 (1.14-6.57)	0.024
History of dental procedures	0.10 (0.01-0.92)	0.078 (0.008-0.743)	0.001
Family member treated for HBV/HCV	12.10 (0.82-310.1)	15.36 (0.63-373.5)	0.003

Multivariable logistic regression, adjusting for potential confounders, identified several independent predictors of hepatitis infection. A sibling history of hepatitis (AOR: 11.68, p=0.001), unsafe injections from informal health care providers (AOR: 5.07, p=0.024), sharing sharp materials (e.g., blades or razors; AOR: 5.58, p=0.018), a history of jaundice or prior HBsAg positivity (AOR: 5.93, p=0.021), and having a family member treated for HBV or HCV (AOR: 15.36, p=0.003) were significantly associated with infection. While a history of dental procedures was associated with lower odds of HBV/HCV infection (AOR: 0.078; 95% CI: 0.008-0.743; p=0.001), this estimate is based on a small number of events and may be unstable. Therefore, this apparent protective effect should be interpreted with caution.

The observed associations indicate that household exposure and unsafe medical practices are important contributors to maternal hepatitis infection. These findings emphasize the need for enhanced antenatal screening and improved immunization coverage to better address hepatitis infection during pregnancy.

## Discussion

This study provides updated hospital-based estimates of HBV and HCV infection among pregnant women in Lucknow, highlighting the ongoing relevance of these infections within antenatal care settings. The observed HBV prevalence of 2.5% aligns with values reported from several regions in northern India, although variability across facilities and populations persists. HCV prevalence was low (0.3%) but consistent with regional studies. Overall, the combined HBV/HCV prevalence of 2.8% among pregnant women in northern India is comparable to prior regional estimates [3,6]. When compared with previous studies, the prevalence reported here aligns with findings from northern India and other developing countries, where HBV prevalence among pregnant women ranges from 1.5% to 3% and HCV from 0.3% to 1% [7,8].

These results support earlier research suggesting that pregnancy generally does not exacerbate hepatic inflammation but requires close monitoring due to the risk of perinatal transmission [9]. Our findings reaffirm the strong association between infection and unsafe injections from informal health care providers, which has been consistently reported in South Asian populations [12]. Women aged ≥25 years had a significantly higher prevalence of infection compared to those aged <25 years (p=0.0159), suggesting possible cumulative exposure risk or cohort-related differences in vaccination or healthcare access. Although prevalence was numerically higher in the first trimester, the difference across trimesters was not statistically significant (p = 0.078), suggesting that infections were likely pre-existing rather than pregnancy-acquired. It highlights the importance of early antenatal screening and timely neonatal prophylaxis to prevent vertical transmission. Multivariable analysis identified unsafe injections from informal health care providers, a history of jaundice or prior HBsAg positivity, and having a sibling with hepatitis as significant independent predictors of infection [12]. These results underscore the continuing role of informal health care providers and unsterile medical practices in hepatitis transmission during pregnancy. Additionally, the association with a family history of hepatitis suggests that both behavioral and environmental factors contribute to viral spread.

These findings highlight the importance of expanding universal antenatal screening, addressing unsafe injection practices, and strengthening awareness through targeted information, education, and communication (IEC) strategies, in line with the NVHCP guidelines [11]. Further studies with larger sample sizes are needed to validate these associations and more accurately quantify risk factors among pregnant women in India.

This study has several important limitations. Although the overall sample size was substantial, the number of HBV and HCV-positive cases (n=56) was relatively small, resulting in sparse outcome data and reduced statistical precision. Consequently, several regression estimates exhibited wide CIs. Information on neonatal immunoprophylaxis was collected descriptively and should not be interpreted as evidence of program effectiveness. Finally, the facility-based sampling approach may restrict the generalizability of these findings to the wider pregnant population of the district.

Key points

HBV and HCV remain important public health concerns among pregnant women, underscoring the continued relevance of antenatal screening in routine maternal care. Socioeconomic vulnerability, household exposure, and unsafe or informal medical practices appear to contribute to maternal infection, reflecting persistent gaps in awareness and access to safe health care services. Most infected women were identified at an early stage of disease, highlighting the opportunity for timely detection through antenatal screening and appropriate clinical linkage. Strengthening antenatal screening services, ensuring timely neonatal hepatitis B prophylaxis, and establishing systematic referral pathways for infected mothers are essential to effective prevention. Overall, integrating hepatitis prevention strategies into routine maternal and child health services is crucial for advancing national and global viral hepatitis elimination goals.

## Conclusions

This facility-based cross-sectional study provides updated prevalence estimates of HBV and HCV among pregnant women in Lucknow and describes the distribution of selected exposure histories within antenatal care settings. Although exploratory associations were observed for certain risk factors, these findings should be interpreted cautiously due to the limited number of infected cases and sparse data in key exposure categories, which may affect the precision of the estimates. Future research incorporating larger numbers of infected mothers, longitudinal infant testing, and population-representative sampling is essential to better quantify transmission risk, validate determinants of infection, and evaluate preventive strategies.

Within these limitations, the study contributes valuable descriptive epidemiological data relevant to antenatal screening programs in northern India and underscores the need for rigorous, adequately powered analytical studies to inform hepatitis prevention efforts. Maternal HBV and HCV continue to represent a significant public health concern in India, reinforcing the importance of sustained antenatal screening and comprehensive perinatal hepatitis prevention strategies to support progress toward the WHO 2030 hepatitis elimination targets.
