# Correction: Seroprevalence and Determinants of Hepatitis B Virus and Hepatitis C Virus Infection Among Pregnant Women in North India: A Cross-Sectional Study

**DOI:** 10.7759/cureus.c405

**Published:** 2026-02-20

**Authors:** Abhishek Yadav, Monika Agarwal, Vikasendu Agarwal, Amita Jain, Sumit Rungta, Prabhaker Mishra, Amit Goel, Anjoo Agarwal, Himanshu Reddy, Priyanka Yadav, Milind Wardhan, Neeraj K Gupta, Manisha Pathak

**Affiliations:** 1 Community Medicine and Public Health, King George's Medical University, Lucknow, IND; 2 Medical and Health Services, State Viral Hepatitis Programme Management Unit, National Viral Hepatitis Control Programme (NVHCP), Lucknow, IND; 3 Microbiology, All India Institute of Medical Sciences, Raebareli, IND; 4 Gastroenterology, King George's Medical University, Lucknow, IND; 5 Biostatistics and Health Informatics, Sanjay Gandhi Postgraduate Institute of Medical Sciences, Lucknow, IND; 6 Gastroenterology, Sanjay Gandhi Postgraduate Institute of Medical Sciences, Lucknow, IND; 7 Obstetrics and Gynaecology, King George's Medical University, Lucknow, IND; 8 Internal Medicine, King George's Medical University, Lucknow, IND; 9 Obstetrics and Gynaecology, Silver Jubilee Community Health Centre (CHC), Lucknow, IND; 10 Child Health Division, National Health Mission, Lucknow, IND; 11 Family Medicine, King George's Medical University, Lucknow, IND; 12 Public Health, Sarsawati Medical College, Lucknow, IND

This article has been corrected to address errors in the interpretation of statistical significance. The Abstract, Results, and Discussion sections have been revised to clarify that the higher prevalence among women aged ≥25 years was statistically significant (p = 0.0159), and that differences in prevalence across trimesters were not statistically significant (p = 0.078). The corrected text now accurately reflects the reported p-values and their corresponding interpretations.

